# Effects of different crop rotation patterns on the growth of Guizhou sorghum and rhizosphere soil quality: implications for agricultural policy

**DOI:** 10.3389/fmicb.2025.1637727

**Published:** 2025-09-03

**Authors:** Manjing Chen, Jiaqi Shen, Ying Zhao, Peiyun Xie, Yingying Zhou, Yuwen Jiang, Xiaofeng Deng, Yan Ren, Mingbo Shao

**Affiliations:** ^1^Institute of Dryland Crops, Guizhou Academy of Agricultural Sciences, Guiyang, China; ^2^School of Public Administration, Guizhou University of Finance and Economics, Guiyang, China; ^3^Renhuai Bureau of Agriculture and Rural Affairs, Zunyi, China; ^4^Guizhou Light Industry Technical College, Guiyang, China; ^5^Guizhou Duyun Liquor Factory Co., Ltd., Duyun, China

**Keywords:** sorghum, continuous cropping obstacle, crop rotation, soil quality, microbial community, sorghum yield

## Abstract

**Introduction:**

Sorghum, an important crop for food and raw material for Baijiu production, is typically cultivated using monocropping. However, the impact of long-term sorghum monocropping on soil physicochemical properties and microbial communities is poorly elucidated. Hence, this study was conducted to evaluate the effects of different crop rotation patterns on sorghum growth and the soil microenvironment in Guizhou Province, China.

**Methods:**

Following a randomized block design, the effects of three cropping patterns, continuous sorghum cropping (SSS), sorghum–rapeseed–sorghum (SRS), and sorghum–stem mustard–sorghum (SMS), on sorghum yield, rhizosphere soil quality, and soil microbial community structure were compared.

**Results:**

Compared with the SSS treatment, the SRS and SMS treatments significantly improved the agronomic traits of sorghum as well as the physicochemical traits of soil. Among the treatments, urease and invertase activities were the highest in SRS. Moreover, compared with the SSS treatment, the SRS and SMS treatments significantly altered the composition of the rhizosphere microbial community by reducing the abundance of pathogenic phyla. Furthermore, correlation analysis revealed that soil organic matter, total potassium, available potassium, available phosphorus, and invertase activity are key environmental factors influencing the soil microbial community structure.

**Discussion:**

Sorghum rotation with other crops, such as rapeseed and stem mustard, can improve the soil microenvironment, regulate the soil microbial community structure, alleviate continuous cropping obstacles, and enhance sorghum quality and yield. This finding highlights the importance of optimizing crop rotation patterns to alleviate continuous cropping obstacles.

## 1 Introduction

As the fifth-largest dryland crop worldwide in terms of yield, sorghum is widely used in feed, brewery, biomass energy, and food production (Diouf, 2016). Sorghum is a major raw material for brewery production in Guizhou’s Baijiu industry, which is worth hundreds of billions of yuan. With the upgrading of the industry and the improvement of sorghum varieties, the planting area and yield of Guizhou sorghum have gradually increased, ranking among the top in China (Wang C. et al., 2022). However, the typical Karst topography of Guizhou poses limitations to the per capita cultivated land area and land resources. Monocropping is implemented in Guizhou to cultivate sorghum for brewing purposes. However, the intensification of agricultural activities and long-term sorghum monocropping aggravate problems with the soil environment (Shao et al., 2023), which is the foundation for crop growth, as high-quality soils provide sufficient nutrients for crops. Long-term continuous cropping significantly reduces the soil aggregate stability, physicochemical properties, and enzyme activities (Alami et al., 2021; Holatko et al., 2024), increases the accumulation of toxic metabolites, salts, and acids (Pervaiz et al., 2020), alters the rhizosphere microbial community structure (Chen X. et al., 2022), modulates the relative abundance of beneficial and pathogenic bacterial and fungal phyla (Zhang et al., 2022), and ultimately reduces the crop quality and yield (Yu et al., 2024).

Crop rotation is an agricultural practice that considers land use and conservation. Reasonable crop rotation balances soil nutrient utilization, prevents soil-borne diseases (Malik et al., 2024), improves soil physicochemical properties, and affects soil microbial community diversity, dominant composition, and complexity in paddy fields (Zhang et al., 2023), thereby regulating soil fertility, enhancing soil productivity, alleviating continuous cropping obstacles caused by single cropping (Wang F. et al., 2022), and increasing yield and profit (Bybee-Finley et al., 2024). Recently, several researchers have investigated soil characteristics and microbial community structures resulting from different crop rotation patterns. They highlight the relationship between crop growth and the soil environment and elucidate that crop rotation can effectively reduce soil bulk density (Fang et al., 2022), enhance the stability of large soil aggregates (Tiemann et al., 2015), increase soil organic carbon storage (Yang L. et al., 2024), and strengthen the connectivity and stability of microbial ecological networks (Holatko et al., 2024), thus enhancing soil quality, reducing external inputs such as fertilizers and pesticides, and maintaining or increasing crop yields (Ma et al., 2003).

Sorghum, a member of the Poaceae family, is generally intolerant to continuous cropping. Li et al., (2024) found that long-term continuous sorghum cropping leads to nutrient imbalance in the rhizosphere, resulting in decreased growth indices and yield. Previous studies have shown that *Sesbania cannabina*–sorghum rotation can enhance soil fertility and structure, promote soil biological activity, and further improve soil quality (Wu et al., 2024). As an important specialty coarse grain crop in Guizhou, a vast planting area is allotted for the cultivation of sorghum for brewing purposes.

Although research on variety breeding, cultivation techniques, and brewing processes of sorghum has been conducted, studies on the impact of long-term continuous sorghum cropping on the soil microenvironment are scarce. Hence, this study was conducted to evaluate the effects of different sorghum rotation patterns on sorghum growth and soil quality. The findings of this study provide theoretical and technical support for alleviating sorghum continuous cropping obstacles and increasing sorghum production in Guizhou.

## 2 Materials and methods

### 2.1 Experimental site and design

Field experiments were conducted from 2023 to 2024 at the Shatian sorghum experimental planting plot in Shanshui Village, Luban Street, Renhuai City, Zunyi City, Guizhou Province (E106°17′, N27°42′). The experimental site is characterized by a subtropical humid monsoon climate, an average annual precipitation of 800–1,000 mm, a flat terrain, an altitude of 915 m, and typical yellow soil. Sorghum was continuously cropped at the experimental site from 2020 to 2023. A uniform planting pattern was adopted: sorghum was planted at a rate of two seeds per hole, with a row spacing of 50 cm, plant spacing of 25 cm, and planting density of 10,700 plants/hm^2^. A single crop was cultivated per year, followed by a fallow period in autumn and winter. On September 28, 2023, before conducting the experiment, soil samples from the 0–20 cm soil layer were collected using the five-point sampling method to measure the following soil parameters: pH (6.68), soil organic matter (SOM, 38.2 g/kg), alkali-hydrolyzable nitrogen (223 mg/kg), available phosphorus (AP, 34.8 mg/kg), and available potassium (AK, 170 mg/kg).

In the experiment, which began in October 2023, three treatments—SSS (continuous cropping of sorghum for 5 years), SRS (sorghum–rapeseed–sorghum rotation after 4 years of continuous sorghum cropping), and SMS (sorghum–stem mustard–sorghum rotation after 4 years of continuous sorghum cropping)—each with three replicates (totaling nine plots, each with an area of 49 m^2^), were arranged in a randomized block design. Fertilization levels were consistent across plots. A 1.0-m isolation zone was established between different plots, and tillage was carried out separately for each plot. Sorghum and other autumn–winter cash crops were planted according to local standards.

Hongyingzi, Youyan 2020, and Yuzao 100 were the tested sorghum, rapeseed, and stem mustard varieties, respectively, in the experiment. Rapeseed was sown in the first 10 days of October 2023 and harvested in late April 2024. Stem mustard was sown in late October 2023 and harvested in mid-January 2024. Sorghum was sown in early May 2024 and harvested in early September 2024.

### 2.2 Sample collection

At the filling stage (August 12, 2024) of sorghum, three sorghum plants were randomly selected from each plot. Using a sterile sampling shovel, the soil-attached root system (diameter: 20 cm; depth: 30 cm) was excavated in a ring along the base of the sorghum stem. The root system was gently lifted and shaken to remove loosely attached soil, while the tightly adhering soil along the growth direction of the root system was brushed off using a sterile soft brush with a width of 2 cm. Mixed rhizosphere soil samples were prepared per plot. After debris, such as dead leaves, gravel, and residual roots, had been removed from the mixed samples using tweezers, the soil samples were passed through a 2-mm sieve. Afterwards, the soil samples were placed in self-sealing bags with labels, quickly frozen in dry ice, and immediately transported to the laboratory. Subsequently, the soil samples were divided into two portions: one portion was stored in an ultra-low temperature refrigerator at −80°C, while the other portion was air-dried for preservation after sieving.

### 2.3 Morpho-anatomical, chemical, and molecular analyses

#### 2.3.1 Sorghum agronomic traits

At the jointing (July 12, 2024) and filling stages (August 12, 2024) of sorghum, five representative sorghum plants were randomly selected from each plot to measure their leaf area, plant height, and stem diameter.

#### 2.3.2 Sorghum root activity

At the jointing stage (July 12, 2024) and filling stages (August 12, 2024) of sorghum, three representative sorghum plants were randomly selected from each plot. Roots within a selected area (width: 40 cm; length: 50 cm; depth 40 cm) were excavated, placed in self-sealing bags with unique numbers, preserved with dry ice, and transported to the laboratory. After carefully washing the roots, root activity was measured using the triphenyltetrazolium chloride method.

#### 2.3.3 Soil physicochemical properties

The refrigerated soil samples were brought back to the laboratory, and their water content was immediately measured to be 32.74 ± 1.42%. The air-dried soil samples were subjected to the following physicochemical analyses: pH determination using a pH meter with a soil:water ratio of 1:2.5 (Liu L. et al., 2022), SOM quantification via chromic acid oxidation–reduction titration (external heating method) (Bremner and Jenkinson, 1960), total nitrogen (TN) estimation via the Kjeldahl method, total phosphorus (TP) measurement via sodium hydroxide alkali fusion–molybdenum–antimony colorimetry, total potassium (TK) determination via flame photometry, alkali-hydrolyzable nitrogen estimation via diffusion absorption, AP determination via 0.5 mol/L sodium bicarbonate extraction–molybdenum–antimony colorimetry, and AK quantification via ammonium acetate–flame photometry (Jiang et al., 2025).

#### 2.3.4 Soil enzyme activities

The invertase (INV) and urease (URE) enzyme activities in the refrigerated soil samples were measured using the micro-method (Frankeberger and Johanson, 1983) and visible spectrophotometry (Tabatabai and Bremner, 1972), respectively.

#### 2.3.5 High-throughput sequencing of soil bacterial 16S and fungal internal transcribed spacer

Total microbial community DNA was extracted using the CretMag™ Power Soil DNA Kit (Suzhou Cretaceous Biotechnology, Jiangsu, China). The V3–V4 variable region of the 16S rRNA gene was amplified via PCR using primers 341F (5′-CCTAYS SSRBGCASCAG-3′) and 806R (5′-GGACTACHVSSSTWT CTAAT-3′). The internal transcribed spacer (ITS) region was amplified using primers ITS1F (5′-CTTGGTCATTTAGAGGAAGTAA-3′) and ITS2R (5′-GCTGCGTTCTTCATCGATGC-3′). The PCR-amplified products were purified, quantified, and processed to construct libraries via the NEXTflex™ Rapid DNA-Seq Kit (Bioo Scientific, Austin, TX, USA). Guizhou Bailuoni Testing Technology Co., Ltd. (Guizhou, China) performed sequencing on a NovaSeq SP PE250 platform (Illumina, San Diego, CA, USA). Microbial composition, abundance, and diversity were analyzed using FLASH, QIIME, and UPARSE software, respectively.

### 2.4 Statistical analysis

Data were sorted and statistically analyzed using Excel. One-way analysis of variance were performed using SMSS 19.0.

## 3 Results and discussion

### 3.1 Effects of different crop rotation patterns on sorghum growth

#### 3.1.1 Plant field performance

**Figure 1:**
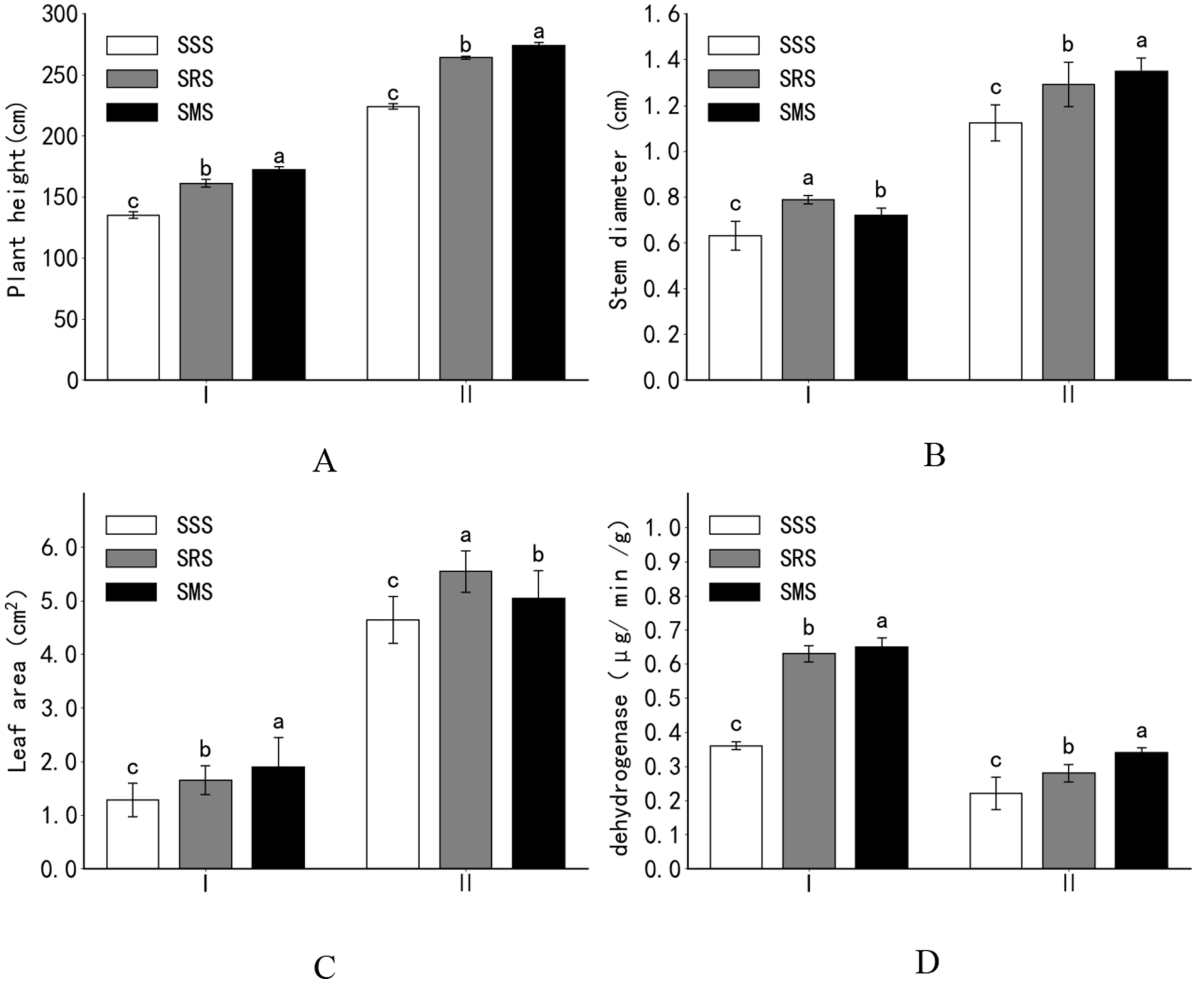
Effects of crop rotation patterns on the growth of sorghum plants (**A**: Plant height; **B**: Stem diameter; **C**: Leaf area; **D**: Dehydrogenase). SSS, continuous sorghum cropping; SRS, sorghum–rapeseed crop rotation; SMS, sorghum–stem mustard–sorghum crop rotation. I, Jointing stage; II, Dough stage. Different small letters at the same growth period indicate significant differences among treatments at *p* < 0.05.

Compared with the SSS treatment, the SRS and SMS treatments significantly promoted sorghum growth (Figures 1A–C). Compared with the plant height, stem diameter, and leaf area of the SSS sorghum plants at the jointing stage, those of the SRS sorghum plants increased by 19, 25, and 29%, respectively, while those of the SMS sorghum plants significantly increased by 27, 14, and 48%, respectively (*p* < 0.05). At the panicle flowering stage, various agronomic traits of the SRS and SMS sorghum plants significantly improved compared with those of the SSS sorghum plants. At the filling stage, the plant height and stem diameter of the SMS sorghum plants were higher than those of the SRS sorghum plants, while the leaf area of the SRS sorghum plants was higher than that of the SMS sorghum plants.

#### 3.1.2 Plant root activity

At the jointing stage, the root activity of the SRS and SMS sorghum plants significantly increased by 75 and 81%, respectively, than that of the SSS sorghum plants (*p* < 0.05). At the panicle flowering stage, the root activity of the SRS and SMS sorghum plants increased by 27 and 55%, respectively, compared with that of the SSS plants. At both stages, the root activity of sorghum was higher in the SMS treatment than in the SRS treatment. Overall, the root activity of sorghum in the SRS and SMS treatments was higher than that in the SSS treatment (Figure 1D).

#### 3.1.3 Plant yield

The yields of the SRS and SMS sorghum plants were higher than those of the SSS sorghum plants (Table 1); specifically, the SRS plants had the highest yield, which was 12% higher than that of the SSS plants (*p* < 0.05). Further analysis of yield components revealed that compared with the SRS and SMS treatments, the SSS treatment significantly decreased the 1,000-grain weight of sorghum grains by 18% (*p* < 0.05), thus affecting the final sorghum yield. Therefore, long-term continuous cropping leads to prominent continuous cropping obstacles in sorghum, ultimately having a significant impact on sorghum yield.

**Table 1:** Effects of crop rotation patterns on sorghum yield and its components.

Treatment	1,000-grain mass (g)	Grain mass per panicle (g)	Yield (kg/hm^−2^)
SSS	18.06 ± 1.1a	32.22 ± 0.8b	403.59 ± 9.4b
SRS	21.98 ± 1.3b	50.16 ± 1.2a	456.37 ± 6.4a
SMS	21.95 ± 0.5b	40.49 ± 1.6b	430.58 ± 7.8a

SSS, continuous sorghum cropping; SRS, sorghum–rapeseed crop rotation; SMS, sorghum–stem mustard–sorghum crop rotation. Different lowercase letters in the same column indicate significant differences among the treatments (*p* < 0.05).

### 3.2 Effects of different crop rotation patterns on the physicochemical properties of sorghum rhizosphere soil

The SOM, TP, TK, and alkali-hydrolyzable nitrogen content of the rhizosphere soil of sorghum in the SRS and SMS treatments were significantly higher than those of the rhizosphere soil of sorghum in the SSS treatment (*p* < 0.05; Table 2). Both SSS and SMS treatments significantly increased soil pH. Meanwhile, the concentrations of AP and AK in the rhizosphere soil of sorghum in the SRS treatment were significantly lower than those in the rhizosphere soil of sorghum in the SSS and SMS treatments (*p* < 0.05). Soil enzymes are important mediators in soil biochemical processes, playing a crucial role in maintaining soil ecological functions. Their activity reflects the ability of soil nutrient transformation and transport, serving as an important basis for evaluating comprehensive soil fertility (He et al., 2024). Among the treatments, URE and INV activities were highest in the SRS treatment; specifically, these significantly increased by 39 and 56%, respectively, compared with those in the SSS treatment (*p* < 0.05). Therefore, the fertility status of sorghum rhizosphere soil is significantly affected by different planting patterns.

**Table 2:** Physicochemical properties of the rhizosphere soil of sorghum in different crop rotation patterns.

Soil physicochemical parameters	Control	Treatment	
	SSS	SRS	SMS
pH	6.29 ± 0.20c	7.47 ± 0.58a	6.70 ± 0.85b
Organic matter (g·kg^−1^)	41.98 ± 0.33a	50.90 ± 1.41a	49.55 ± 1.49a
Total nitrogen (g·kg^−1^)	2.53 ± 1.14a	2.99 ± 0.85a	2.98 ± 0.72a
Total phosphorus (g·kg^−1^)	1.41 ± 1.35b	1.47 ± 0.46b	1.72 ± 0.25a
Total potassium (g·kg^−1^)	25.90 ± 0.27b	26.70 ± 0.30b	27.30 ± 0.42a
Available nitrogen (mg·kg–^1^)	165.00 ± 4.45a	166.00 ± 2.52a	169.00 ± 2.52a
Available phosphorus (mg·kg–^1^)	100.90 ± 17.7b	71.70 ± 0.71c	151.60 ± 1.78a
Available potassium (mg·kg–^1^)	438.00 ± 5.80a	211.00 ± 5.75c	420.00 ± 4.98b
Urease (μg·g^−1^·d^−1^)	414.20 ± 13.69b	575.60 ± 4.73a	365.10 ± 07.6c
Invertase (μg·g^−1^·d^−1^)	31.40 ± 1.59b	43.07 ± 1.67a	26.51 ± 1.16c

SSS, continuous sorghum cropping; SRS, sorghum–rapeseed crop rotation; SMS, sorghum–stem mustard–sorghum crop rotation. Different lowercase letters indicate significant differences among the treatments in the same soil layer (*p* < 0.05).

### 3.3 Effects of different crop rotation patterns on the diversity of microbial communities in sorghum rhizosphere soil

#### 3.3.1 Number of operational taxonomic units

**Figure 2:**
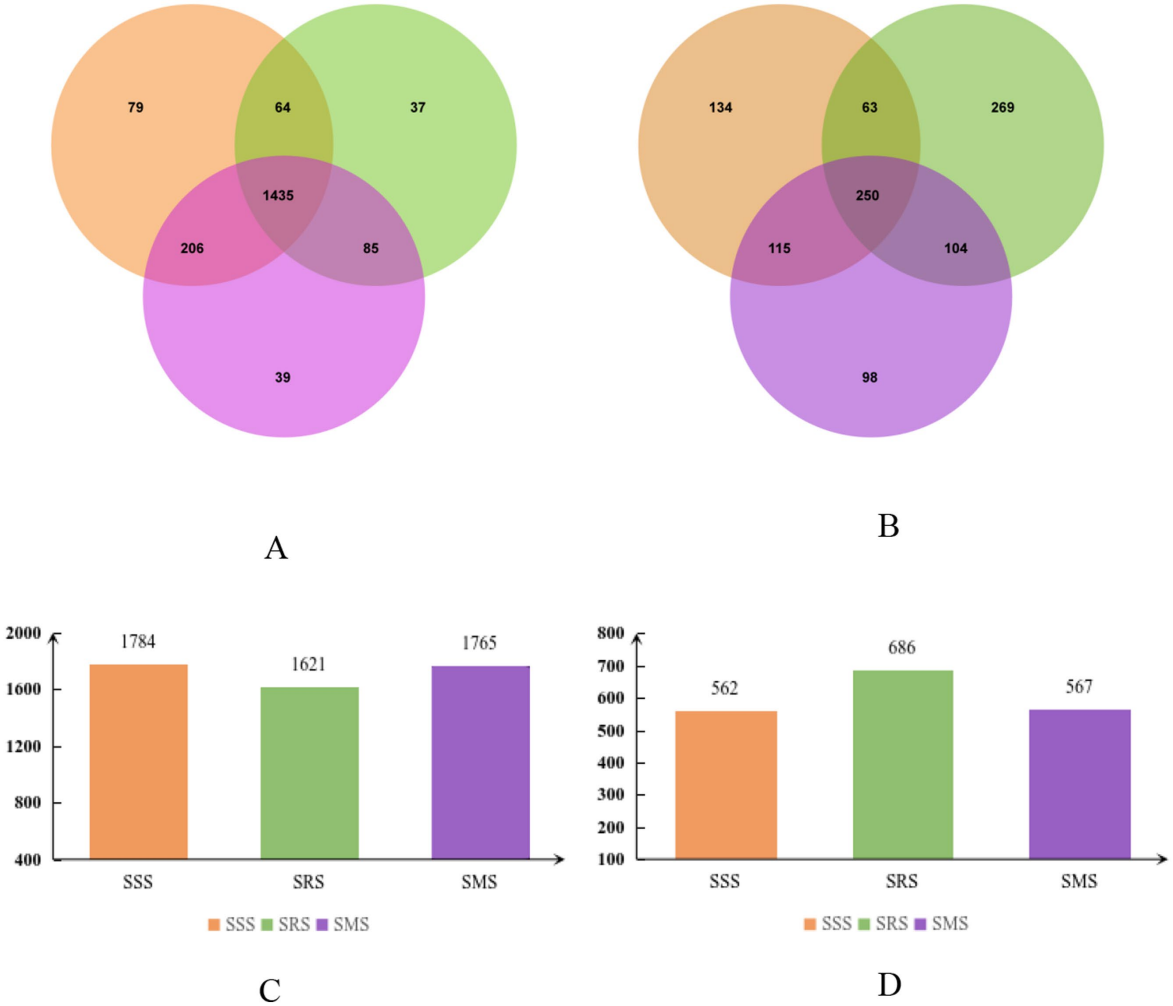
Venn diagram showing the number of operational taxonomic units (OTUs) of soil bacteria **(A,C)** and soil fungi **(B,D)** in sorghum rhizosphere soil samples from different planting patterns.

Venn diagrams can be used to count the shared and unique operational taxonomic unit (OTU) numbers of microorganisms in different treatment samples, intuitively showing the distribution of microbial communities under different treatments (Fouts et al., 2012). Figure 2 shows the number of unique and shared bacterial and fungal OTUs in the rhizosphere soil of sorghum in different treatments, as a result of metagenomic deep sequencing analysis and Venn diagram analysis of OTUs in different samples. Among the treatments, the SRS soil had the lowest number of bacterial OTUs (1,621), which was 9.1 and 8.2% lower that than in the SSS and SMS soils, respectively (Figures 2A,C). In contrast, the SRS soil had the highest number of fungal OTUs (686; Figures 2B,D). The number of bacterial and fungal OTUs did not significantly differ between the SSS and SMS soils (*p* > 0.05). Therefore, the SRS planting pattern has a greater impact on the structure of the sorghum rhizosphere soil microbial community.

#### 3.3.2 Alpha diversity of soil microbial communities

**Figure 3:**
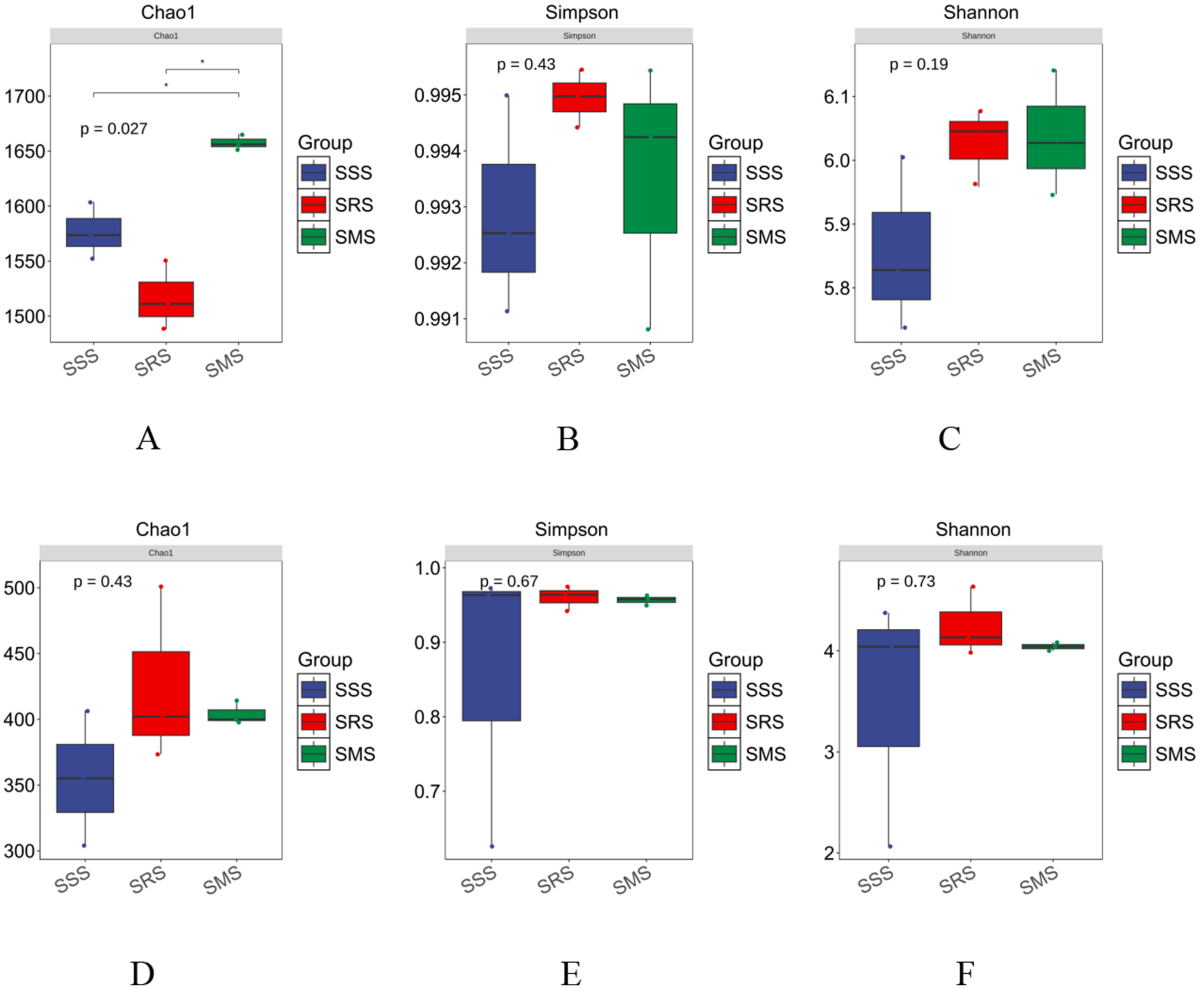
Alpha diversity of soil microbe of sorghum in different planting patterns. Alpha diversity of bacteria **(A–C)**; alpha diversity of fungi **(D–F)**. * indicates a significant difference at *p* < 0.05.

Alpha diversity in community ecology reflects the abundance and diversity of microbial communities. The Chao1 index represents the richness index, while the Shannon and Simpson indices estimate microbial diversity. The Chao1 index of the bacterial community in the SMS soil was significantly higher than that in the SSS and SRS soils (*p* < 0.05); in particular, the Chao1 index of the bacterial community in the SRS soil was the lowest (*p* < 0.05) (Figure 3A). The Shannon and Simpson indices of the bacterial communities in the SRS and SMS soils were higher than those in the SSS soil (*p* > 0.05) (Figures 3B,C), with the highest Simpson index recorded in the SRS soil. Meanwhile, the Chao1, Shannon, and Simpson indices of the fungal communities in the SRS and SMS soils were higher than those of the fungal community in the SSS soil; however, the differences were not significant (*p* > 0.05) (Figures 3D–F). These results indicate that the SRS and SMS rotation patterns affect the abundance and diversity of bacterial and fungal communities in sorghum rhizosphere soil; specifically, the SMS pattern elicits a greater impact on bacterial community abundance, while the SRS pattern influences both fungal community abundance and diversity.

#### 3.3.3 Beta diversity of soil microbial communities

**Figure 4:**
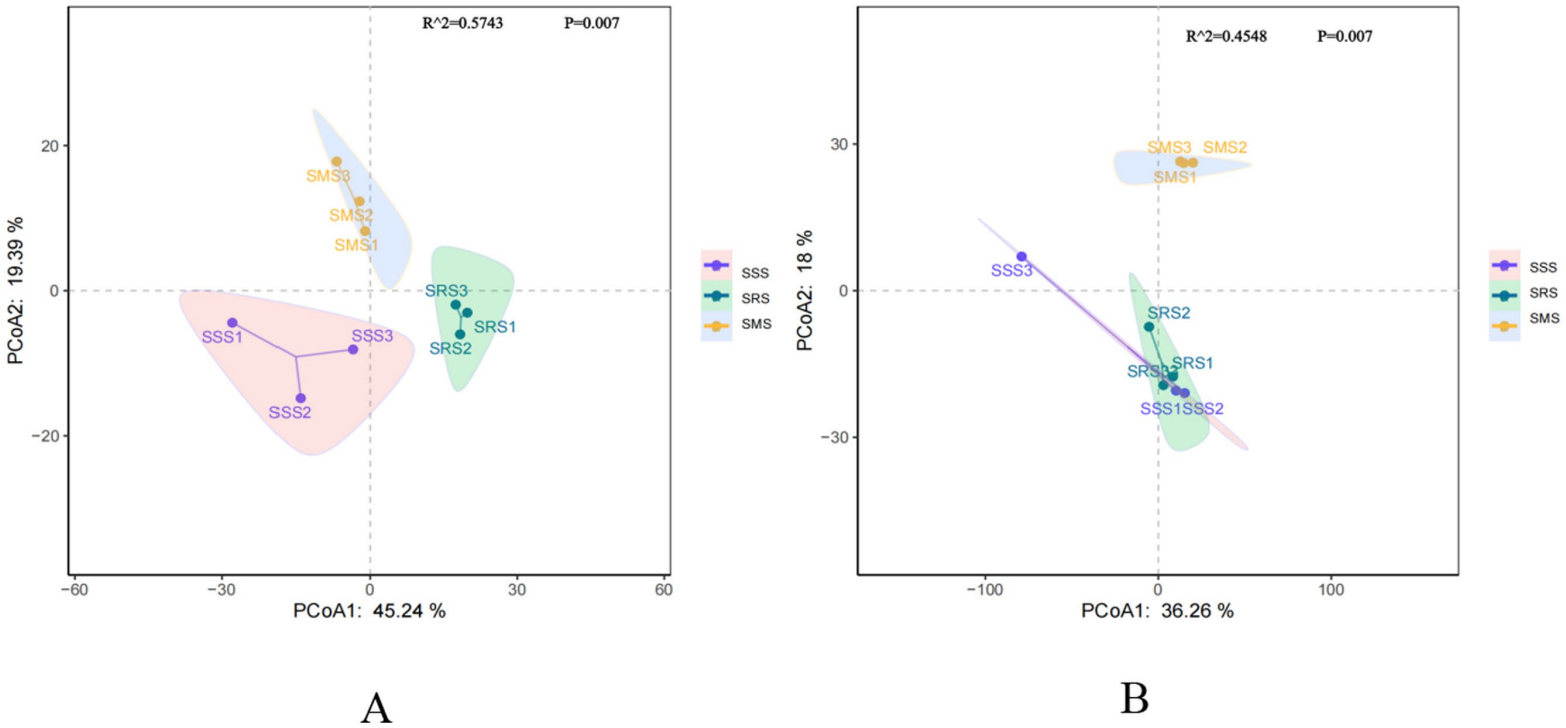
Principal coordinate analysis (PCoA) of rhizosphere soil bacterial **(A)** and fungal **(B)** communities in different sorghum rotation patterns.

Principal coordinate analysis (PCoA) is a classic non-constrained ordination method (Classical Multidimensional Scaling, cMDScale) (Wang Y. et al., 2022). It projects the sample distance matrix into a low-dimensional space while maximizing the retention of original distance relationships, accurately reflecting the beta diversity differences of soil microbial communities among different samples. PCo1 and PCo2 explained 45.24 and 19.39% of the bacterial community structure variation, respectively, clearly separating the bacterial communities in the three treatments (Figure 4A). Anosim analysis revealed significant differences in soil bacterial community composition among the treatments (Adonis R^2^ = 0.5743; *p* = 0.007). Meanwhile, PCo1 and PCo2 explained 36.26 and 18% of the fungal community structure variation, respectively (Figure 4B). The fungal communities in the SSS and SRS soils were relatively clustered, while those in the SMS soil were separated along the PCo2 axis. Anosim analysis revealed significant differences in soil fungal community composition among the treatments (Adonis R^2^ = 0.4548; *p* = 0.007). These results indicate that different planting patterns are the primary drivers of changes in sorghum rhizosphere microbial communities.

#### 3.3.4 Structure of soil bacterial and fungal communities

##### 3.3.4.1 Soil bacterial structure

**Figure 5:**
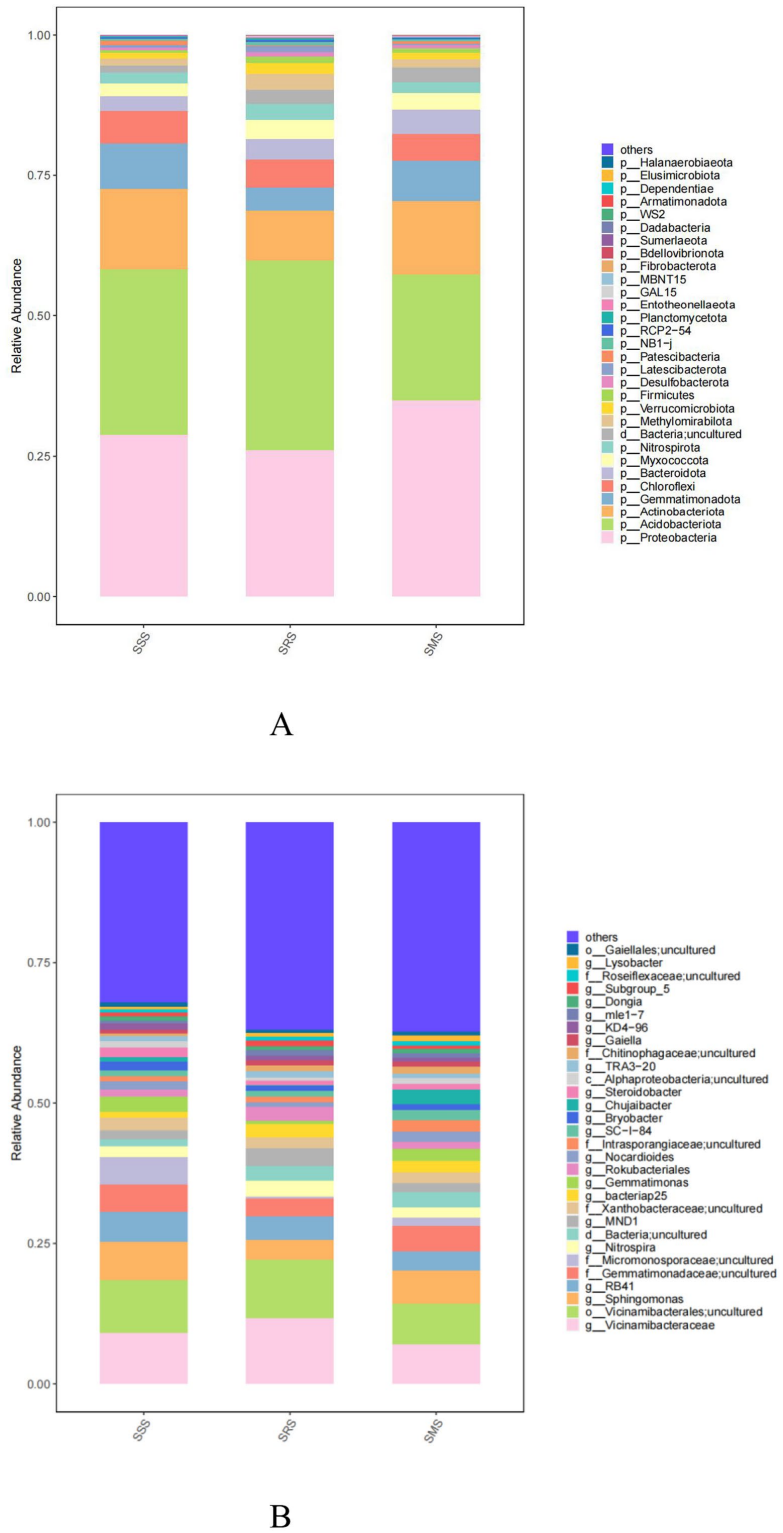
Bacterial community structure at the phylum **(A)** and genus levels **(B)** in sorghum rhizosphere soil in different planting patterns.

The five dominant bacterial phyla in the SSS, SRS, and SMS rhizosphere soils were Proteobacteria, Acidobacteriota, Actinobacteriota, Gemmatimonadota, and Chloroflexi (Figure 5A). The relative abundance of Proteobacteria in the SSS soil (28.71%) was lower than that in the SMS soil (34.91%) but higher than that in the SRS soil (26.04%). The relative abundance of Acidobacteriota was highest in the SRS soil (33.86%) and lowest in the SMS soil (22.48%). The relative abundances of Actinobacteriota, Gemmatimonadota, and Chloroflexi across the treatment soils were 8.76–14.38%, 4.16–7.94%, and 4.78–5.82%, respectively.

Meanwhile, the five most abundant genera were *Vicinamibacterales*, *Vicinamibacteraceae*, *Sphingomonas*, *RB41*, and *Gemmatimonadaceae*; their relative abundances were 9.51–10.42%, 8.97–11.66%, 3.56–6.81%, 3.38–5.24%, and 3.15–4.54%, respectively (Figure 5B). The relative abundances of *Vicinamibacterales* and *Vicinamibacteraceae* were highest in the SRS soil; specifically, these were higher by 0.9 and 2.7 percentage points than those in the SSS soil. Similarly, the relative abundances of the other three dominant genera were highest in the SRS soil, exceeding those in the SRS and SMS soils by 0.09–0.32, 0.11–0.18, and 0.04–0.17 percentage points, respectively.

##### 3.3.4.2 Soil fungal community structure

**Figure 6:**
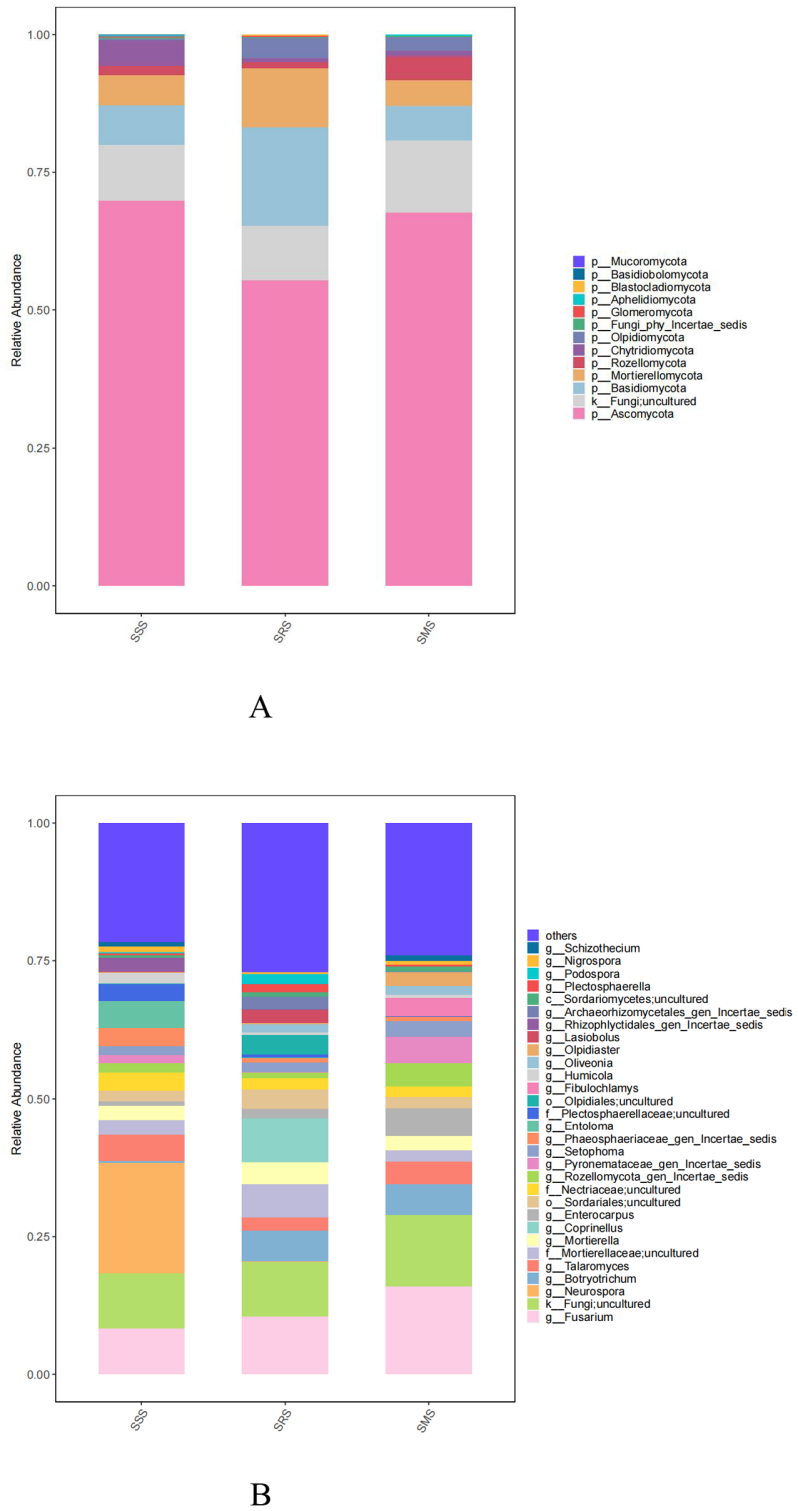
Fungal community structure at the phylum **(A)** and genus levels **(B)** in sorghum rhizosphere soil in different planting patterns.

The five dominant fungal phyla across the treatment soils were Ascomycota, unclassified_k_Fungi, Basidiomycota, Mortierellomycota, and Rozellomycota (Figure 6A). The relative abundance of Ascomycota was highest in the SSS soil (69.85%); meanwhile, it ranged from 55.39 to 67.69% in the SRS and SMS soils. Among the treatment soils, Basidiomycota and Mortierellomycota were most abundant in the SRS soil (17.78 and 10.69% relative abundance, respectively), whereas unclassified_k_Fungi and Rozellomycota were most abundant in the SMS soil (12.61 and 4.29% relative abundance, respectively).

Meanwhile, the five most dominant fungal genera in the treatment soils were *Fusarium*, *unclassified_k_Fungi*, *Talaromyces*, *Neurospora*, and *Mortierellaceae* (Figure 6B). *Neurospora* (20% relative abundance) was only present in the SSS soil, while *Botryotrichum* (5.50–5.58% relative abundance) was one the dominant genera in the SRS and SMS soils.

### 3.4 Effects of soil physicochemical properties on soil microbial community structure

#### 3.4.1 Correlation between soil physicochemical properties and dominant soil microorganism phyla

**Figure 7:**
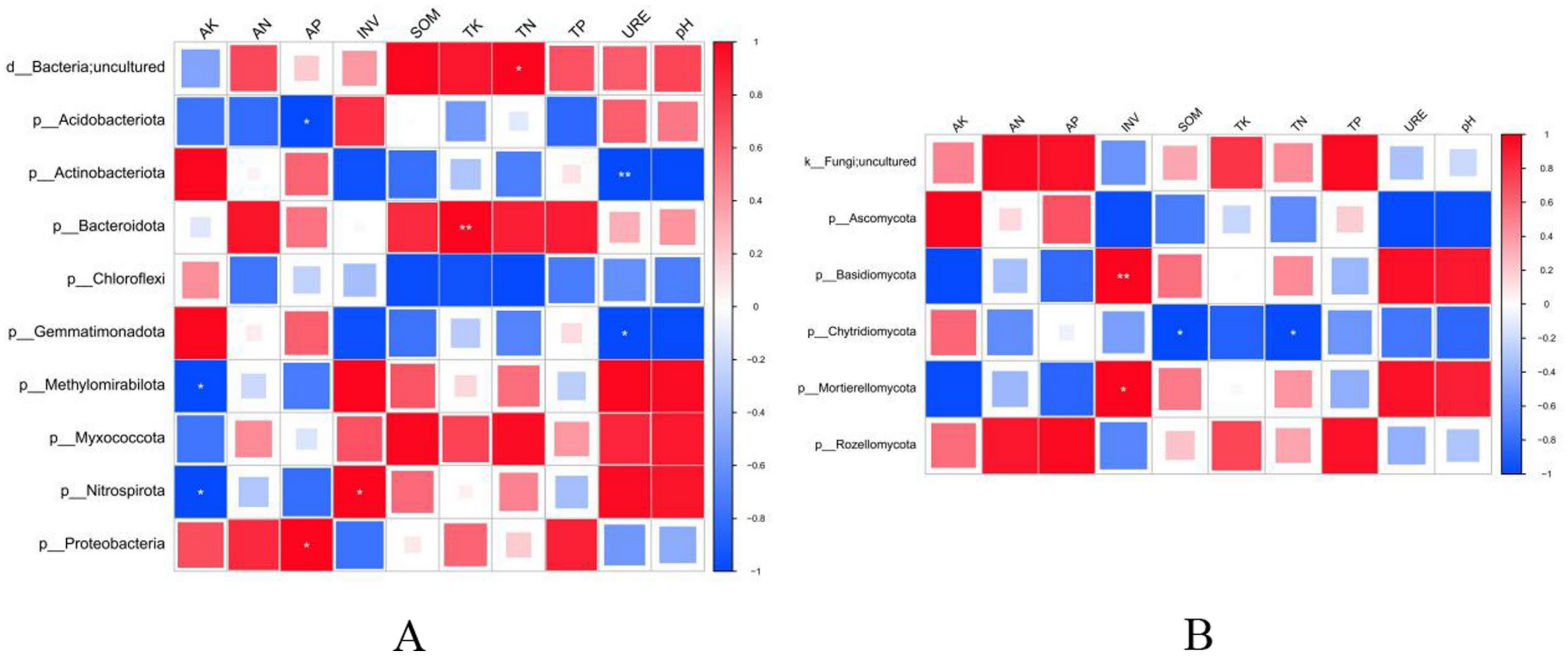
Correlation analysis between soil physicochemical properties and dominant bacterial **(A)** and fungal **(B)** phyla. AK, available potassium; AN, available nitrogen; AP, available phosphorus; INV, invertase; SOM, soil organic matter; TK, total potassium, TN, total nitrogen; TP, total phosphorus; URE, urease. * indicates a significant correlation at *p* < 0.05; ** indicates a significant correlation at *p* < 0.01.

To analyze the relationship between soil biological characteristics and soil nutrients under different sorghum planting patterns, correlation analysis was performed between soil physicochemical properties and dominant bacterial phyla using analytical software. As shown in Figure 7A, the relative abundances of Methylomirabilota and Nitrospirota were significantly negatively correlated with AK. The relative abundance of Acidobacteriota was significantly negatively correlated with AP, while that of Proteobacteria was significantly positively correlated with AP. A strong and significant positive correlation was observed between the relative abundance of Bacteroidota and TK. The relative abundance of the unclassified phylum was significantly positively correlated with TN, while that of Nitrospirota was significantly positively correlated with INV. The relative abundance of Actinobacteriota exhibited a strong and significant positive correlation with URE, while that of Gemmatimonadota was significantly negatively correlated with URE.

Meanwhile, correlation analysis between soil physicochemical properties and the dominant fungal phyla (Figure 7B) revealed a strong and significant positive correlation between the relative abundance of Basidiomycota and INV. The relative abundance of Mortierellomycota was significantly positively correlated with INV, while that of Chytridiomycota was significantly negatively correlated with both SOM and TN.

#### 3.4.2 Redundancy analysis between soil physicochemical properties and dominant soil microorganism phyla

**Figure 8:**
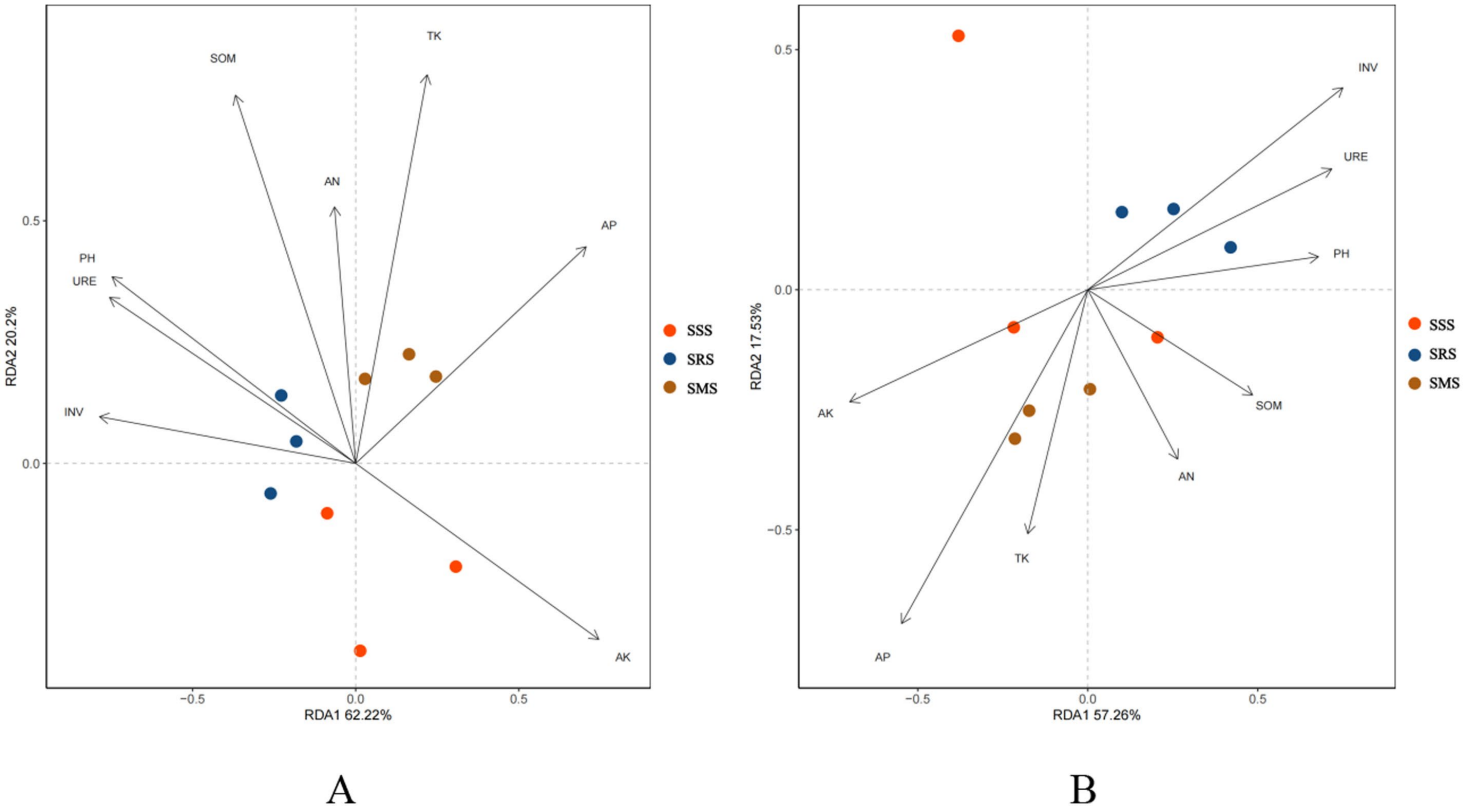
Redundancy analysis between the physicochemical properties of sorghum rhizosphere soil and soil bacteria genera **(A)** and fungi genera **(B)**.

Redundancy analysis (RDA) was performed using the microbial community structure and soil physicochemical properties under different sorghum planting patterns. For the soil bacterial community structure (Figure 8A), the first ordination axis (RDA1) and the second ordination axis (RDA2) explained 82.42% of the environmental factors affecting the microbial community among the different treatments in cumulative variance. For the soil fungal community structure (Figure 8B), the first and second axes explained 74.79% of the environmental factors, indicating reliable analysis results. Further analysis showed that SOM, TK, and AK are the main environmental factors influencing the soil bacterial community structure, while INV and AP are the main environmental factors influencing the soil fungal community structure.

## 4 Discussion

### 4.1 Crop rotation improves soil nutrients and enzyme activities

Continuous cropping obstacles, also known as replant disease, refer to the phenomenon where long-term continuous cultivation of the same crop or its related species on the same land leads to changes in soil nutrient availability, allelopathy, and soil microorganisms, thereby affecting crop growth (Ma et al., 2023; Zeeshan Ul Haq et al., 2023) Long-term monocropping disrupts the soil ecological environment (Chen et al., 2020), induces pest infestations (Xi et al., 2019), and ultimately impairs crop growth and reduces yield, exerting a significant negative impact on agricultural development. Numerous studies have shown that crop rotation can improve soil physicochemical properties and, thus, promote crop growth. For example, maize–soybean rotation increases soil carbon and nitrogen storage, thereby improving soil quality (Luo et al., 2024), maize–tobacco rotation increases soil pH and cation exchange capacity (Jiang et al., 2025), and pea–potato rotation enhances SOM and TN content (Wang et al., 2023). In this study, we evaluated the effects of sorghum–rapeseed and sorghum–stem mustard rotations on the growth of Guizhou sorghum, and the results revealed that compared with traditional continuous sorghum cropping, the quality of the rhizosphere soil of sorghum significantly improved after sorghum–rapeseed and sorghum–stem mustard rotations, as evidenced by increases in soil pH and SOM, TP, TN, TK, and alkali-hydrolyzable nitrogen levels, because rapeseed and stem mustard increase soil nutrient content and the likelihood of colonization by various microorganisms through rhizodeposition and residue return (Zhou et al., 2024). Consequently, nitrogen, phosphorus, potassium, and other nutrients are released from the soil through mineralization and humification, providing a material basis for the continuous replenishment of the soil nutrient pool (Guggenberger, 2005). In this study, the AP and AK levels in the rhizosphere soil of sorghum in the SRS treatment were significantly lower than those in the rhizosphere soil of sorghum in the SSS and SMS treatments, probably because rapeseed is a phosphorus- and potassium-loving crop. Although its roots can activate insoluble phosphorus and potassium through chelation, given consistent fertilization levels, it still absorbs large amounts of phosphorus and potassium from the soil to maintain normal plant growth (Urricariet et al., 1995; Zhao et al., 2020). These traits of rapeseed provide support for the field management of sorghum–rapeseed rotation.

Soil URE and INV serve as key catalysts in nitrogen and carbon cycles between the soil and plants (Gu et al., 2009; Qin et al., 2022) investigated diversified tobacco rotation patterns and found that flue-cured tobacco rotation effectively increased soil URE and INV activities. Similarly, our results revealed that compared with the SSS treatment, URE and INV activities in the rhizosphere soil of sorghum significantly enhanced after the SRS treatment, probably due to the specific substances secreted by rapeseed roots, such as flavonoids and glucosinolates, that can effectively regulate and improve the structure and activity of soil microbial communities, thereby increasing soil enzyme activities. For example, glucosinolates attract nitrifying bacteria, which form symbiotic relationships with microorganisms that produce soil enzymes such as URE and phosphatase; hence, glucosinolates not only provide soil bacteria with a suitable growth environment but also indirectly enhance soil enzyme activities (Dong et al., 2018).

### 4.2 Crop rotation remodels and regulates functional microbial communities

Diversified crop rotation systems increase the species richness and diversity of soil microorganisms, making a more complex and stable soil microbial community structure (Sun et al., 2024), which in turn regulates and improves the soil environment. The results of this study revealed that compared with the SSS treatment, the SRS and SMS treatments affected the abundance and diversity of rhizosphere soil microorganisms to varying degrees. The significant increase in the abundance of bacterial communities as well as the diversity of soil bacteria and fungi in sorghum rhizosphere soil in the SMS treatment may be attributed to the unique physiological characteristics of stem mustard roots and their exudates. As a succulent plant with high water content, stem mustard is more easily decomposed than fibrous plants. Under sufficient moisture conditions, more and diverse beneficial microorganisms quickly gather on the plant and decompose its nutrients. The SRS treatment also improved the diversity of bacteria and fungi in sorghum rhizosphere soil; however, unlike in previous studies, the ratio of bacteria to fungi decreased in the SRS treatment owing to the abundance of organic substances such as cellulose and lignin in rapeseed plants. After rapeseed harvest, residual root stubble and aboveground residues are returned to the soil, inducing changes in the abundance of soil bacteria and fungi. The results of this study are consistent with a previous finding that as the complexity of crops increases, beneficial fungi responsible for decomposing plant residues exhibit stronger adaptability while bacterial abundance decreases sharply (Miao et al., 2025).

The relative abundance of the dominant bacteria and fungi varied among the treatments. Notably, the SRS treatment effectively regulated the abundance of Ascomycota in the fungal community by significantly reducing its relative abundance. Studies have shown that some Ascomycota species are plant pathogens that cause root rot and affect plant growth (Challacombe et al., 2019). In contrast, the SRS treatment increased the relative abundances of Basidiomycota and Mortierellomycota, probably due to the interaction between rapeseed root exudates, such as sugars, proteins, and mucilage, and soil nutrients to provide abundant carbon sources and energy for beneficial fungi, attracting them to gather and reproduce around the roots. In addition, Basidiomycota and Mortierellomycota elicit antagonistic effects on some plant pathogens, inhibiting pathogen growth and competing for more nutrients for their own reproduction (Card et al., 2021).

Several researchers have reported that different soil factors affect the distribution of microbial structures. For example, Philippot et al. (2024) identified that soil pH, soil organic carbon, and oxygen partial pressure are key drivers of microbial community structure and activity, while Sadeghi et al. (2023) used the Cubist model to predict soil microbial communities based on soil properties and found that soil nutrients, such as nitrogen, phosphorus, potassium, and organic matter, are significantly correlated with the relative abundance of most soil microorganisms. The results of correlation and redundancy analyses in this study showed that INV activity, TP, TK, AK, and SOM are key factors affecting the structure of microbial communities in sorghum rhizosphere soil; specifically, these were significantly positively correlated with the relative abundances of Proteobacteria, Bacteroidota, Basidiomycota, and Nitrospirota. These findings indicate that sorghum rotation can significantly increase soil nutrient content and enzyme activity, thereby increasing the abundance of beneficial microbial communities and improving soil quality.

### 4.3 Crop rotation drives root–shoot synergy and yield improvement

The optimization of soil properties further reshapes the rhizosphere microenvironment, and a favorable rhizosphere microenvironment can directly drive the structural succession and functions of microbial communities. Ultimately, the “root–shoot interaction” mechanism promotes the overall growth performance of sorghum, effectively alleviating the impact of continuous cropping, enhancing plant growth, and significantly increasing crop yield (Zhang et al., 2024). For example, Wu et al. (2025) demonstrated that highland barley–wheat or highland barley–rapeseed rotation improved soil quality, thereby increasing highland barley yield. Zhou et al. (2024) found that optimizing the rice–rapeseed rotation system significantly improved crop yield and yield stability, as rice and rapeseed yields increased as the duration of rotation prolonged. Yang X. et al. (2024) reported that diversified wheat rotation increased crop yield. Healthy roots usually secrete a large amount of organic and antibacterial substances into the soil, attracting various microorganisms to enrich the rhizosphere. These secretions serve as a bridge connecting plant roots and rhizosphere microorganisms, playing a key role in influencing the structure of the rhizosphere biological community (Haichar et al., 2008). The results of this study showed that compared with the SSS treatment, the SRS and SMS treatments significantly increased sorghum root activity. The optimization of soil properties further reshapes the rhizosphere microenvironment, creating favorable conditions for sorghum growth. This may be because long-term continuous cropping increases soil bulk density, reduces total soil porosity, leads to soil compaction, and restricts root growth (Hammel, 1989; Ajayi et al., 2021). Rotation with taproot crops, such as rapeseed and stem mustard, can effectively improve soil physical structure, enhance soil permeability and aggregate stability (He et al., 2024), and increase soil water retention through two-season cultivation (Schlegel et al., 2019), making the soil looser and promoting root development. Long-term continuous cropping easily leads to an imbalance in the soil microbial community structure, resulting in the proliferation of pathogens, reduction of beneficial bacteria, and accumulation of allelochemicals in crops (Ding et al., 2024; Gan et al., 2025), thereby inhibiting healthy root development. Root exudates, such as organic acids secreted by rapeseed roots, can activate insoluble phosphorus in the soil (Luo et al., 1999), and rapeseed rhizosphere soil is rich in potassium-solubilizing bacteria that dissolve insoluble potassium (Xiao et al., 2017). This nutrient enrichment effect can directly alleviate the problem of single soil nutrients due to continuous sorghum cropping, providing a more balanced nutrient environment for root development. After stem mustard straw is returned to the field, the decomposed organic matter and available nutrients accelerate the degradation of sorghum autotoxic substances in continuous cropping soil (Chen S. et al., 2022), reducing allelopathic inhibition and promoting root growth.

In our study, compared with the SSS treatment, the SRS and SMS treatments significantly improved the field agronomic traits of sorghum, such as plant height, stem diameter, and leaf area, and significantly increased sorghum yield. This finding is attributed to the enhanced sorghum root system in rotation treatments through improved nutrient supply and reduced allelopathic pressure (Chen Y. et al., 2022). Sorghum root exudates regulate the root microbial community structure, improve the efficiency of soil nutrient absorption and utilization (Xu et al., 2025), and promote aboveground growth through the root–shoot feedback mechanism (Liu X. et al., 2022). For example, at the jointing and panicle flowering stages, the leaf area of sorghum in the rapeseed rotation area increased by 48 and 19%, respectively, compared with that in the SSS treatment. The photosynthetic pigment content and photosynthetic rate of leaves may have significantly increased, improving the synthesis rate of photosynthetic products. Increased plant height and stem diameter resulting from the SRS and SMS treatments promote the transport of assimilates from leaves to sorghum panicle grains, making the grains fuller and ultimately improving grain quality and yield.

## 5 Conclusions and policy recommendations

This study demonstrates that crop rotation is an effective cultivation measure to improve the rhizosphere soil microenvironment of sorghum. SRS rotation significantly enhanced soil nutrients, microbial structural diversity, and sorghum root activity, agronomic traits, and yield. The results indicate that sorghum rotation alters the soil microbial community structure by increasing the abundance of Proteobacteria, Acidobacteriota, Basidiomycota, and Mortierellomycota while effectively reducing the abundance of pathogenic phyla, such as Ascomycota. These changes are related to the root exudates of different crops and the improvement of soil environmental factors through crop straw returning, which promotes microbial activity and nutrient availability. Improved soil nutrients and a better root growth environment led to a significant increase in sorghum yield. These findings highlight the importance of optimizing interactions among planting structures, soil, and microorganisms, and underscore the potential of diversified crop rotation systems in improving soil microenvironment and alleviating continuous cropping obstacles. Future research should continue to explore the effects of different crop rotations on sorghum soil health and microbial functions as well as their application effects in various sorghum planting systems. Based on the findings of the study we recommend the following agricultural policies to alleviate continuous cropping obstacles.

### 5.1 Optimize planting patterns and promote crop rotation systems

To optimize sorghum planting patterns, rotation demonstration bases should be established in major sorghum-producing areas in Guizhou (e.g., Renhuai and Zunyi), and farmers should be guided in adopting the two-year three-crop rotation patterns of sorghum–rapeseed and sorghum–stem mustard to replace traditional continuous cropping. To address the needs of the local Baijiu industry, the combination of brewing sorghum and characteristic cash crops should be promoted, and farmers’ income should be augmented through industrial chain extension (e.g., rapeseed processed into edible oil or stem mustard developed into pickled products). Moreover, clarify the connection time of rotation crop sequences (e.g., rapeseed is sown in October and harvested in April, while sorghum is sown in May), optimize fertilization and plant protection plans, reduce difficulty in farmers’ operation, and ensure the standardization and replicability of rotation patterns.

### 5.2 Strengthen soil management and promote precision cultivation

To improve the soil nutrient characteristics of rotation areas, implement the technology of soil testing and formula fertilization + organic fertilizer return to field, focus on supplementing phosphorus and potassium fertilizers, and improve soil carbon and nitrogen reserves by combining crop straw return to field to improve soil physical and chemical properties. Install long-term soil microorganism monitoring points in main producing areas to dynamically track the changes in microbial community structure (e.g., bacteria/fungi ratio, dominant microbial communities), providing data support for rotation effect evaluation. Furthermore, develop a soil health digital platform to achieve precise recommendations for rotation patterns and fertilization plans through farmers’ uploads of sample test results.

### 5.3 Deepen scientific research–industry collaboration and improve policy support

Encourage scientific research institutes to conduct research on root interaction mechanisms of rotation crops, analyze the regulatory effects of root exudates of different crops on soil pathogens, and explore more efficient rotation combinations (e.g., sorghum paired with leguminous crops) to provide theoretical support for optimizing planting structures. Guide Baijiu enterprises (e.g., Moutai and Xijiu) must sign rotation orders with farmers and enhance farmers’ enthusiasm through price incentives (e.g., premium purchasing). Include rotation in the cultivated land protection project, provide subsidies to farmers who implement rotation for a long time, and prioritize farmland infrastructure construction to ensure the sustainability of the farming system.

## Data Availability

The data presented in the study are deposited in the NCBI BioProject repository, accession number PRJNA1301879.
